# Analysis on the plane and mechanism of tongue-originated obstruction in Obstructive Sleep Apnea Syndrome (OSAS) patients with macroglossia

**DOI:** 10.1016/j.bjorl.2025.101709

**Published:** 2025-09-13

**Authors:** Min Huang, Guohao Chen

**Affiliations:** a Department of Otolaryngology, the First Affiliated Hospital, Fujian Medical University, China; b Department of Otolaryngology, National Regional Medical Center, Binhai Campus of the First Affiliated Hospital, Fujian Medical University, Fuzhou, China; c Fujian Institute of Otolaryngology, the First Affiliated Hospital, Fujian Medical University, Fuzhou, China

**Keywords:** OSAS, Macroglossia, Tongue/anatomy & histology, MRI, PSG

## Abstract

**Objective:**

To investigate the plane and related mechanisms of tongue-originated obstruction in Obstructive Sleep Apnea Syndrome (OSAS) patients with macroglossia, utilizing pharyngeal Magnetic Resonance Imaging (MRI) in combination with Upper Airway Pressure Monitoring (UAPM).

**Methods:**

Nineteen OSAS patients with macroglossia and 19 healthy controls were enrolled in this study. Every case was examined by pharyngeal MRI and Polysomnogram (PSG). The minimum Retropalatal Space (RP), the minimum Retrolingual Space (RL), soft palate thickness and the minimum space from the dorsal tongue to the soft palate (TP) were measured on a midsagittal plane. UAPM was measured only in the OSAS group. The differences of results between groups were statistically analyzed.

**Results:**

Both UAPM and pharyngeal MRI indicated that the obstruction plane was present in patients with OSAS along the velopharyngeal plane. The mean values of both RP and RP/RL of patients with OSAS in this study were significantly smaller than control group values (*p* < 0.05). There was no significant difference in RL and soft palate thickness across the two groups (*p* > 0.05). The value of TP in both groups was close to 0, showing no significant difference between groups (*p* > 0.05).

**Conclusions:**

The velopharyngeal plane was the most common site demonstrating obstructive etiology in OSAS patients with macroglossia. The related mechanism is suspected to include the backward movement of the soft palate caused by the high arch and the fall of the middle segment of the hypertrophic tongue. These findings may inform tongue-related surgical site decision-making for OSAS patients with macroglossia.

**Level of evidence:**

IV: Retrospective cohort study.

## Highlights

• The velopharyngeal plane was the most common site to develop obstruction.
• Compared with the normal, retrolingual space was increased in the OSAS patients.
• The dorsal tongue in contact with the soft palate during supine sleep for everyone.
• Analysis of the mechanisms of tongue- originated obstruction in OSAS patients.
• The choice of the surgical site is in the middle part of the tongue for OSAS.

## Introduction

Obstructive Sleep Apnea Syndrome (OSAS) arises from a combination of anatomical upper airway narrowing and impaired neuromuscular compensation during sleep, leading to recurrent airflow limitation.^1^ It represents an independent risk factor for hypertension, coronary heart disease, diabetes, etc.,^2^ OSAS is also an important cause of sudden nocturnal death and road traffic accidents. Therefore, more attention has been paid to the treatment of OSAS. Currently, many strategies have been developed to treat OSAS, among which CPAP (continuous positive airway pressure) treatment is recognized as the first-line treatment.^3^^,^^4^ However, it is estimated that 30%‒50% of patients remain unable to accept or tolerate.^5^ As such, the AASM (American Academy of Sleep Medicine) holds the view that surgery is a fundamental treatment modality.^6^ Uvulopalatopharyngoplasty (UPPP) and Lateral Pharyngoplasty are some of the earliest and most widely used surgical procedures for OSAS. Historically, the success rate of isolated soft palate surgeries (e.g., UPPP) for symptomatic relief in unselected OSAS patients has been reported as less than 40%,^7^ though outcomes may improve with patient stratification and multimodal approaches. Related studies have shown that the obstruction plane was present in most patients with OSAS along the velopharyngeal plane.^8^ Friedman et al. reported that the rate of symptomatic relief in Friedman stage Ⅲ OSAS patients who received UPPP alone was only 8.1%.^9^ While severe OSAS patients who received the combination of UPPP and Coblation channeling for the tongue was 15%.^10^ Most tongue-related surgeries focus on the base of the tongue and fail to fully consider and treat the obstruction caused by hypertrophic tongue, which may account for poor surgical results. Moreover, the mechanism of velopharyngeal plane obstruction caused by hypertrophic tongue has not yet been clarified.

If the plane and the mechanism of the obstruction can be correctly determined before surgery, the likelihood of surgical success may be increased. However, determination of the obstruction plane is challenging. Currently, there is no gold standard examination method that can accurately determine obstruction plane. Clinical judgment is consequently based on a comprehensive analysis from a series of examinations. Upper Airway Pressure Monitoring (UAPM) is the only method that can reflect dynamic changes of the obstruction plane, but it cannot determine etiology. Pharyngeal MRI can simultaneously observe the obstruction of different planes in the upper airway and analyze the local anatomy of the obstruction plane. MRI provides high resolution of soft tissues (allowing morphometric investigation) while avoiding radiation exposure. Drug-Induced Sleep Endoscopy (DISE) has emerged as a critical tool for dynamically assessing upper airway collapse patterns (e.g., anteroposterior, concentric) during near-physiological sleep, complementing static imaging modalities like MRI. But DISE requires drug-induced sleep, which may increase patient risks (e.g., respiratory depression) and requires additional equipment and operations.

In the present study, dynamic analysis of UAPM in combination with static observation of pharyngeal MRI was performed to observe tongue-based obstruction in OSAS patients with macroglossia. Simultaneously, the mechanism of obstruction was investigated. This study aimed to provide new insights for site selection of tongue-related surgery, a targeted treatment strategy to improve surgical effectiveness.

## Methods

### Subjects

This study was divided into 2 groups: OSAS group with macroglossia and the normal control group.

Retrospective analysis was performed on 19 subjects (17 males, 2 females) treated at the first affiliated hospital of Fujian Medical University for moderate and severe OSAS with macroglossia from January 2020 to December 2023. The degree of hypertrophy was determined according to the Friedman Tongue Position (FTP) tongue body score standard. All OSAS patients with FTPIII or IV were considered as hypertrophic tongue.^11^, ^12^, ^13^ To obtain relevant anthropometric matching normal controls, a total of 19 volunteers, including 17 males and 2 females, were enrolled in this study. All volunteers were excluded from OSAS by PSG procedures (Table 1). All subjects underwent detailed medical history and laryngological examination. The study obtained informed consent from the patient or guardian for participation in this study. All research procedures received the approval from the institutional medical ethics committee.

**Table 1:** Anthropometric and polysomnographic data of all study subjects.

Group	Number	Gender	Age (y’s)	Height (m)	Weight (kg)	BMI (kg/m^2^)	AHI (times/hour)	LSaO_2_ (%)	ODI (times/hour)
OSAS	19	17 males; 2 females	35 [28, 45]	1.71 [1.70, 1.76]	76 [70, 80]	25.95 [24.06, 27.49]	46.40 [31.40, 52.70]	74 [66, 81]	44.8 [28.1, 51.4]
Control	19	17 males; 2 females	35 [32, 43]	1.71 [1.65, 1.75]	73 [65, 75]	23.99 [23.05, 26.09]	0.9 [0.5, 2.1]	92 [91, 94]	0.9 [0.5, 1.8]
*Z*-walue	‒	‒	−0.015	−0.675	−1.656	−1.854	−5.274	−5.198	−5.274
p-value	‒	‒	1.00^b^	0.506^b^	0.103^b^	0.065^b^	0.000^a^	0.000^a^	0.000^a^

BMI, Body Mass Index; AHI, Apnea/Hypopnea Index; LSaO_2_, Lowest Oxygen Saturation; ODI, Oxygen Desaturation Index.

a *p* < 0.05.

b *p* > 0.05.

The OSAS group inclusion criteria were as follows: 1) Aged between 20–60 years old, no gender limitation; 2) Confirmed diagnosis of OSAS according to the AASM International Classification of Sleep Disorders (ICSD-3)^14^; 3) The tongue body score ≥ grades 3 according to FTP;^15^ 4) No history of medication that affected airway caliber (e.g., sedatives, hypnotics, muscle relaxants, antipsychotics).

The control group inclusion criteria were as follows: 1) Aged between 20–60 years old, no gender limitation; 2) AHI values < 5; 3) No history of medication that affected airway caliber (e.g., sedatives, hypnotics, muscle relaxants, antipsychotics).

All subjects exclusion criteria were as follows: 1) Contraindications for MRI examination, such as metal foreign body implantation, claustrophobia, etc.; 2) Serious cardiopulmonary diseases such as cardiac dysfunction, asthma and chronic obstructive pulmonary disease; 3) Structural abnormality of the nasopharynx; 4) Tonsil grading system ≥ size 3,^11^ 5) Lingual Tonsil Hypertrophy ≥ grade 3;^16^ 6) A history of acute upper respiratory tract infection in the preceding month; 7) Pituitary adenoma and hypothyroidism; 8) Regular or recent use of CPAP.

### Research procedure

#### The tongue body score: Friedman Tongue Position (FTP)

The FTP assesses the resting posture of the tongue within the oral cavity, where the patient is not necessitated to protrude or manipulate the tongue in any manner. During the evaluation of FTP, the healthcare professional should instruct the patient to maximalize mouth opening at least five times, enabling the observer to determine the most consistent and reproducible location of the tongue.

FTP I: The soft palate, pharyngeal arches, uvula, and hard palate are visible. FTP II: The soft palate, uvula, and hard palate are visible. FTP III: Only the soft palate and hard palate are visible. FTP IV: Only the hard palate is visible.

#### PSG and judgment

Both OSAS group and the control group were monitored overnight (more than 6-hs) using a polysomnography monitor (Alice 5 System, 0f Weikang Company, USA). The results were calculated by certified sleep technicians according to the manual subgraph, and were analyzed according to AASM guidelines. The diagnosis of OSAS was performed and graded. Depending on the grading, an AHI value from 5 to 14.9 is classified as mild, 15–30 as moderate, and >30 as severe OSAS.^17^ The control group of the study constituted subjects with AHI values < 5.

#### Pharyngeal MRI and measurement (Table 2)

**Fig. 1:**
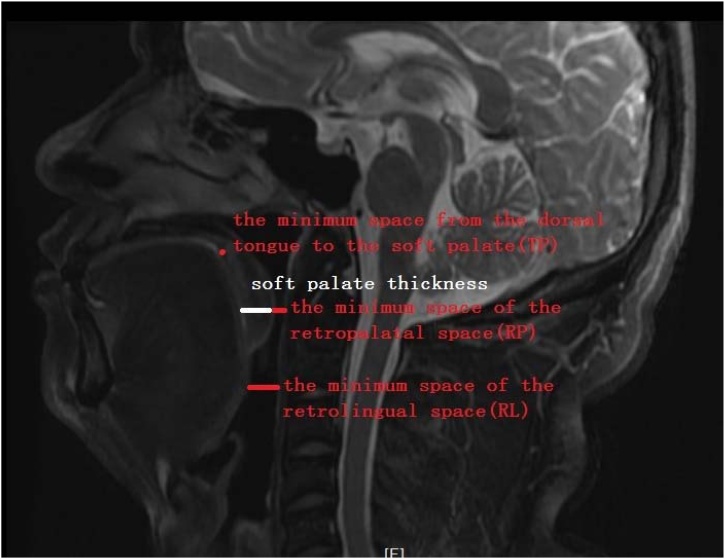
Measurement method of the Pharyngeal MRI.

All subjects underwent pharyngeal MRI 1.5 T Magnetic Resonance Imaging (Philips Achieva, from Netherlands, 1.5 T MRI scanner) at our hospital. All subjects were placed in the supine position with slightly backward tilt (sleep-like position). During the scanning procedure, all subjects were asked to be tight lipped and retain calm breathing. The pharynx was scanned continuously, and the image was reconstructed after scanning. The study observed the retropalatal space and the retrolingual space. Retropalatal space was measured as the plane of the hard palate to the free edge of the uvula; retrolingual space was measured as the free edge of the uvula to the superior edge of the epiglottis; soft palate thickness was measured at level of the minimum space of the retropalatal space. On the midsagittal plane of MRI, the following measurements were made: the minimum space of the Retropalatal space (RP), the minimum space of the Retrolingual space (RL), soft palate thickness, and the minimum space from the dorsal tongue to the soft palate (TP); to observe the relationship between the tongue and the soft palate (Measurement method: Fig. 1).

**Table 2:** Pharyngeal MRI morphometrics between the control and OSAS group.

Measurement parameters	OSAS (*n* = 19)	Control (*n* = 19)	*Z*-value	p-value
The minimum space of the Retropalatal space (RP) (cm)	0.36 [0.21, 0.42]	0.53 [0.46, 0.71]	−3.711	0.000^a^
The minimum space of the Retrolingual space (RL) (cm)	1.12 [0.76, 1.37]	1.07 [0.80, 1.32]	−0.599	0.563^b^
RP/RL	0.26 [0.22, 0.47]	0.62 [0.39, 0.68]	−3.243	0.001^a^
The minimum space from the dorsal Tongue to the soft Palate (TP) (cm)	0 [0, 0]	0 [0, 0]	−1.000	0.795^b^
Soft palate thickness (cm)	0.98 [0.88, 1.12]	0.91 [0.84, 1.03]	−1.783	0.075^b^

a *p* < 0.05.

b *p* > 0.05.

#### Upper Airway Pressure Monitoring (UAPM) and analysis

The Apnea Graph (AG200, MRA company, Britain) was used to monitor nocturnal sleep for over 6-h on the OSAS group. Two miniature pressure sensors and two thermal sensors were located on the pressure tube, which could be automatically started according to the set time. The pressure changes in the nasopharynx, retropalatal space, retrolingual space and mid-esophagus were recorded continuously throughout the night (6 h). The collected data was further analyzed by Apnea analysis software for obtaining the types of obstruction and the proportion of obstruction above and below the velopharyngeal plane (Table 3).

**Table 3:** Analysis of Upper Airway Pressure Monitoring (UAPM).

Classification	Upper obstruction dominance	Lower obstruction dominance	*Z*-value	p-value^a^
Obstructive apnea times	0.87 [0.75, 0.94]	0.11 [0.05, 0.22]	−3.744	0.000
Obstructive hypopnea times	0.82 [0.73, 0.91]	0.18 [0.09, 0.23]	−3.823	0.000
Mixed times	0.75 [0, 0.87]	0.11 [0, 0.25]	−2.951	0.003

a *p* < 0.05.

### Statistical analysis

SPSS 25.0 statistical packages were implemented to carry out the statistical analysis. Due to the small sample size of the study, non-parametric tests were used for statistical analysis. All results were expressed as median values [5 quantile; 95 quantile] (M [P5; P95]). For inter-group comparisons, each value was compared by Mann-Whitney *U* test. For intra-group comparisons, each value was compared by Wilcoxon signed-rank test. The test standard of all statistical methods was α = 0.05, where a value of *p* < 0.05 indicated statistical significance.

## Results

The OSAS group with macroglossia included 17 males and 2 females with a mean of age 35 [28, 45] years, height of 1.71 [1.70, 1.76](m), weight of 76 [70, 80](kg), and BMI of 25.95 [24.06, 27.49](kg/m^2^). The control group included 17 males and 2 females with a mean of age 35 [32, 43] years, height of 1.71 [1.65, 1.75], weight of 73 [65, 75](kg), and BMI of 23.99 [23.05, 26.09](kg/m^2^). There were no significant differences in gender, age or BMI between the two groups.

The results of UAPM in the OSAS group indicated that the obstruction plane was mainly localized along the velopharyngeal plane.

There was no difference between groups regarding the minimum Retrolingual space (RL) (*p* > 0.05). It indicated that the tongue base of all the OSAS groups did not fall back.

The mean minimum space of the Retropalatal space (RP) and RP/RL was smaller in the OSAS group compared to controls (*p* < 0.05). It indicated that obstruction plane measured by pharyngeal MRI was mainly localized to the velopharyngeal plane in the OSAS groups. These findings were also consistent with upper airway pressure monitoring.

Soft palate thickness showed no difference between the two groups (*p* > 0.05), indicating that the soft palate in OSAS patients had no obvious thickening and that for the etiology for velopharyngeal plane obstruction is not likely related to soft palate thickness.

In the context of the minimum space from the dorsal Tongue to the soft Palate (TP), the two groups were both close to 0 with no significant difference (*p* > 0.05). This supported that the dorsal tongue was closed to the soft palate for all subjects during supine sleep.

## Discussion

With technological progress and the continuous updating of surgical approaches, numerous treatment modalities have been utilized for OSAS. The CPAP is clearly considered as the first-line treatment but other methods, such as modified oral appliances, weight loss, and hypoglossal nerve stimulation, have also been clinically implemented. Ultimately, surgical therapy still plays a critical role in the treatment of OSAS.^18^ The localization of the obstruction plane and the analysis of its etiology pre-surgically form the primary basis for upper airway surgical site selection and treatment modality. Anatomical and etiological insights are thus crucial to the surgical success.

The obstruction plane of OSAS can be divided into three categories: nasopharyngeal plane, oropharyngeal plane (including velopharyngeal plane and glossopharyngeal plane) and laryngopharyngeal plane.^19^ The oropharyngeal plane is the only muscular-based elastic tube in the upper airway that lacks bone or cartilage stent. One study reported that 30%‒50% of adult OSAS cases are located in the oropharyngeal plane.^20^ The tongue, as the principal soft-tissue in the mouth, is a muscular soft-tissue organ that occupies most of the volume of the oropharyngeal cavity, and can be divided into two parts: the body of tongue and the base of tongue. Both parts are bounded by a terminal sulcus at the back of the tongue, forming a V-shaped groove opening forward. The body of tongue occupies the first 2/3 of tongue, including the tip of the tongue. The base of tongue occupies the posterior 1/3 of tongue and is fixed to the hyoid bone by the lingual muscle.

In the present study, the FTP as the primary criterion for assessing tongue hypertrophy (inclusion threshold: FTP ≤ Grade III), as it directly reflects the dynamic impact of tongue volume and position on airway space and has been strongly correlated with OSAS severity in prior studies.^11^, ^12^, ^13^ Although the modified Mallampati classification is widely used for pre-anesthesia airway risk assessment, it may be influenced by non-tongue factors, such as the height of the palatine arch, the length of the uvula, or micrognathia, leading to insufficient oral space and causing the tongue to appear “relatively hypertrophic” within the limited space. Such anatomical variations may obscure the true definition of tongue hypertrophy and consequently affect the analysis of the obstructive mechanism. The study applied strict exclusion criteria (such as structural abnormality of the nasopharynx, tonsil grading system ≥ grade 3) to minimize such confounding factors as much as possible. Future research should combine three-dimensional imaging to further distinguish the interaction between true tongue hypertrophy and jaw development abnormalities, in order to more precisely guide surgical decision-making.

**Fig. 2:**
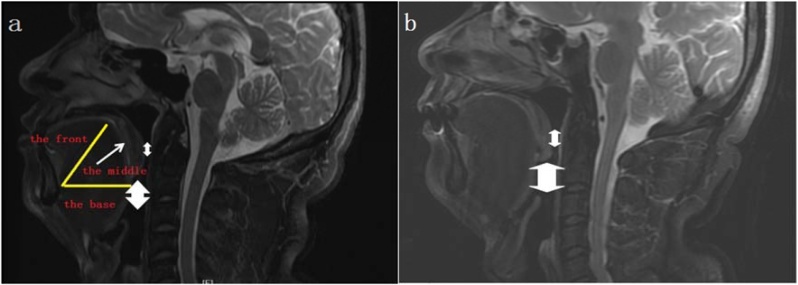
Midsagittal MRI of OSAS patients (a) and normal controls (b) at the velopharyngeal plane. The middle part of the tongue is attached to soft palate in both groups. However, only Figure (a) shows backward movement of the soft palate, resulting in collapse of the velopharyngeal plane.

In the present study, there was no difference between groups regarding the minimum Retrolingual space (RL). It indicated that the tongue base of all the OSAS groups did not fall back. The tongue base hypertrophy doesn’t mean reduced retrolingual space. The mean minimum space of the retropalatal space and RP/RL was smaller in the OSAS group compared to controls. It indicated that obstruction plane measured by pharyngeal MRI was mainly localized to the velopharyngeal plane in the OSAS groups. The results of UPAM indicated that obstruction plane was mainly localized to the velopharyngeal plane, but not the glossopharyngeal plane. The results of the two analysis were consistent, confirming the mainstream view that retropalatal space is smaller than the retrolingual space in OSAS patients.^21^ Based on the imaging features of the contact between the middle part of the tongue and the soft palate in Fig. 2, it can be inferred the dorsal tongue was high-arched (morphological abnormality) during supine sleep in the two groups. In the context of the minimum space from the dorsal Tongue to the soft Palate (TP), the two groups were both close to 0 with no significant difference. The finding indicated that the dorsal tongue was closed to the soft palate for both OSAS patients and control patients during supine sleep. However, only in OSAS patients caused retropalatal narrowing, resulting in poor ventilation (Fig. 2a and b). Furthermore, soft palate thickness showed no difference between the two groups, indicating that the soft palate in OSAS patients had no obvious thickening and that for the etiology for velopharyngeal plane obstruction is not likely related to soft palate thickness. Hence, we speculate that the tongue base did not fall back in OSAS patients with macroglossia. On the contrary, due to the high arch in the middle part of the tongue, once the tongue is hypertrophic, it is more likely to fall back than the tongue base and to oppress the backward movement of the soft palate, causing retropalatal narrowing. However, Pavel Kavcic et al.^22^ reported that the soft palate in OSAS patients was attached to the tongue base, synchronously moving backward and compressing the airway.

At present, there is no definite conclusion on the mechanism of velopharyngeal plane obstruction caused by hypertrophic tongue. However, it is certain that velopharyngeal plane affects the occurrence and development of OSAS through certain physiological and anatomical mechanisms. Unlike prior studies focusing on tongue base hypertrophy, our findings identify the middle of the tongue as a critical contributor to retropalatal collapse in OSAS patients with macroglossia. This distinction suggests that surgical interventions targeting the middle of tongue (e.g., midline glossectomy) may offer superior outcomes compared to traditional tongue base reduction, particularly when combined with skeletal assessments. Although pharyngeal MRI was performed in awake subjects, which may not fully replicate airway dynamics during sleep and does not characterize the collapse pattern (e.g., anteroposterior, concentric). In future studies, we are incorporating DISE could provide dynamic, real-time insights into collapse patterns. Despite these limitations, the consistency between MRI and UAPM data supports the validity of our static-dynamic correlation analysis. DISE remains the gold standard for dynamic pattern assessment, and future studies should integrate both modalities for comprehensive analysis.

**Fig. 3:**
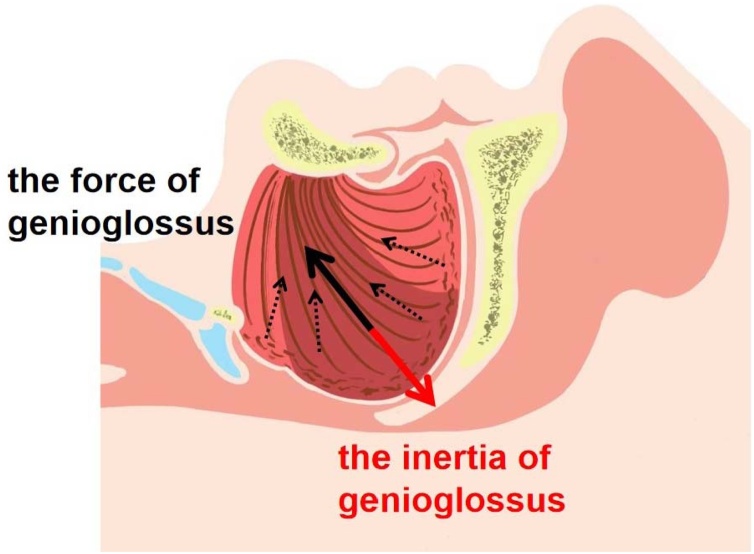
The force of genioglossus.

**Fig. 4:**
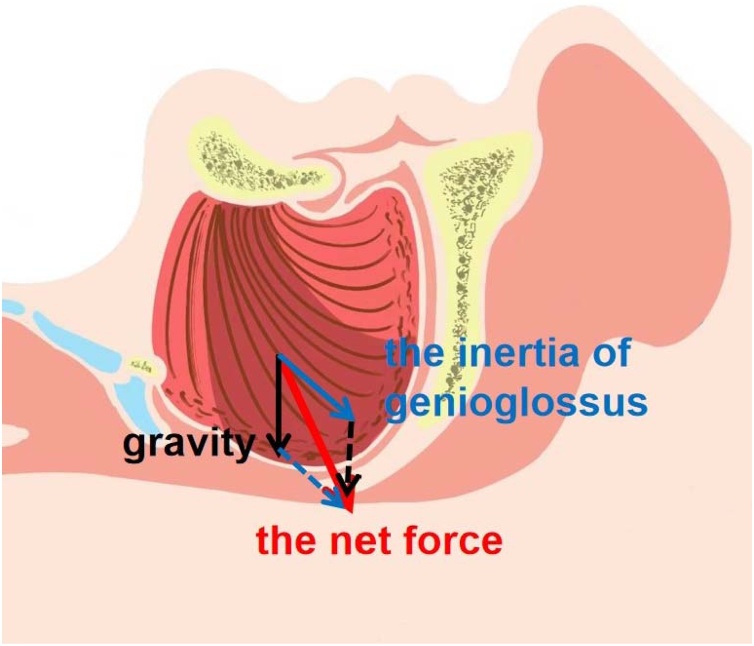
Combined with sleep position and gravity.

The tongue consists of extra-lingual and intro-lingual muscles surrounded by fat and fibrous tissue. The genioglossus is the major muscle that moves the tongue forward. Genioglossus arises from the mandibular mental spine, and the muscle fibers are fan-shaped and scatter backward and upward, most of which stop on both sides of the mid-line of the tongue. According to the principles of muscle contractility and the vector synthesis principle of the force, the ending point of the muscle should move towards the starting point. The action point and the resultant force are thereby mainly located in the middle of the tongue and point downwards, expanding the pharyngeal cavity and stabilizing the upper airway (Fig. 3). Because of its vital functions, genioglossus is called the “safety muscle” of the upper airway. In OSAS patients, genioglossus activity is frequently heightened during wakefulness as a compensatory mechanism to maintain airway patency. However, this compensatory effort diminishes during sleep, particularly in REM stages, contributing to airway collapse.^23^ Under the normal sleeping state with supine position, the gravity of the tongue and the pulling force of genioglossus reach a balance, so that the tongue will not collapse due to the effects of gravity, ensuring the patency of the upper airway. Therefore, we speculate that for OSAS patients with macroglossia, with the aggravation of hypoxia, muscle compensation cannot normally offset the airway load. Due to the sleep-phase-dependent diminution of compensatory tone in genioglossus, the middle part of the tongue acts in the opposite direction because of its inertia (Fig. 3). Combined with sleep position and gravity (Fig. 4), it oppresses the soft palate to block the upper airway, resulting in frequent collapse of the velopharyngeal plane. According to the results of this study, it is challenging to analyze and explain the causes of this obstruction. This theory remains a speculative hypothesis. Further studies are needed to clarify the biomechanical interplay between tongue morphology and soft palate dynamics.

The study’s sample size and single geographic region (East Asia) limits the generalizability of findings. Future multicenter studies with larger and multiethnic cohorts are needed to validate these results.

## Conclusions

In conclusion, this study found that the velopharyngeal plane was the most common site to develop obstruction in OSAS patients with macroglossia, not the glossopharyngeal plane. The related mechanism likely involves the backward movement of the soft palate caused by the high arch and the fall of the middle part of hypertrophic tongue. These findings may provide new insights for tongue-related surgical site decision-making. For OSAS patients with macroglossia, the choice of the surgical site should be in the middle part of the tongue, rather than the base of the tongue or the soft palate. The surgical purpose should focus on enlarging the retropalatal space, not the retrolingual space according to our findings. Consequently, without a clear understanding of the obstruction plane and its etiology, blind implementation of UPPP or the expansion of retrolingual space may contribute to poor surgical efficacy.

## Funding

This work was supported by The 2022 Special Fiscal Fund (BPB-2022CGH).

## Declaration of competing interest

The authors declare no conflicts of interest.
